# Pathogenic and commensal *Escherichia coli* from irrigation water show potential in transmission of extended spectrum and AmpC β-lactamases determinants to isolates from lettuce

**DOI:** 10.1111/1751-7915.12234

**Published:** 2014-12-09

**Authors:** Patrick M K Njage, Elna M Buys

**Affiliations:** Department of Food Science, University of Pretoria Lynwood RoadPretoria, 0002, South Africa

## Abstract

There are few studies on the presence of extended-spectrum β-lactamases and AmpC β-lactamases (ESBL/AmpC) in bacteria that contaminate vegetables. The role of the production environment in ESBL/AmpC gene transmission is poorly understood. The occurrence of ESBL/AmpC in *Escherichia coli* (*n =* 46) from lettuce and irrigation water and the role of irrigation water in the transmission of resistant *E. coli* were studied. The presence of ESBL/AmpC, genetic similarity and phylogeny were typed using genotypic and phenotypic techniques. The frequency of β-lactamase gene transfer was studied in vitro. ESBLs/AmpC were detected in 35 isolates (76%). Fourteen isolates (30%) produced both ESBLs/AmpC. Prevalence was highest in *E. coli* from lettuce (90%). Twenty-two isolates (48%) were multi-resistant with between two and five ESBL/AmpC genes. The major ESBL determinant was the CTX-M type (34 isolates). DHA (33% of isolates) were the dominant AmpC β lactamases. There was a high conjugation efficiency among the isolates, ranging from 3.5 × 10^−2^ to 1 × 10^−2^ ± 1.4 × 10^−1^ transconjugants per recipient. Water isolates showed a significantly higher conjugation frequency than those from lettuce. A high degree of genetic relatedness between *E. coli* from irrigation water and lettuce indicated possible common ancestry and pathway of transmission.

## Introduction

*Escherichia coli* is a leading cause of bacterial infections, foodborne diarrhoeal disease and extraintestinal infections in both humans and animals (Da Silva and Mendonça, 2012; Tadesse *et al*., 2012). *Escherichia coli* strains are found as normal commensals in the intestinal tracts of animals and humans, whereas other strains are important intestinal and extraintestinal pathogens (ExPECs) (Smet *et al*., 2008). In contrast to diarrhoeic strains, ExPECs cause disease in body sites outside the gastrointestinal tract, such as urinary tract infections, neonatal meningitis, sepsis, pneumonia and surgical site infections, as well as infections in other extraintestinal locations (Smith *et al*., 2007).

The impact of *E. coli* on morbidity, mortality and healthcare has not been considerable in the past due to effective antibiotic (AB) therapy (Da Silva and Mendonça, 2012). However, this situation has rapidly changed with the increased acquisition of AB resistance by *E. coli* strains (Da Silva and Mendonça, 2012). β-lactams have been widely and effectively used in human and veterinary medicine to treat *E. coli* infections (Smet *et al*., 2008). β-lactamases, which are bacterial enzymes, inactivate β-lactam ABs by hydrolysis (Shah *et al*., 2004; Moubareck *et al*., 2007). Production of β-lactamase has complicated the treatment of nosocomial infections in Gram-negative pathogens. To overcome the production of β-lactamases, extended-spectrum or third-generation cephalosporins were designed. However, *E. coli* and some other members of the Enterobacteriaceae family are able to produce mutant forms of the ‘older’ β-lactamases referred to as extended-spectrum β-lactamases (ESBLs), which are capable of hydrolysing the new-generation cephalosporins and aztreonam (Wiegand *et al*., 2007). Extended-spectrum β-lactamases are class A β-lactamases consisting of the three main families TEM, SHV and CTX-M (Paterson and Bonomo, 2005), as well as the cephalosporin-hydrolysing group 2de OXA enzymes from class D (Pitout *et al*., 1997; Bush and Jacoby, 2010). AmpC β-lactamases are closely similar to but distinct from ESBLs. In contrast to ESBL producers, AmpC β-lactamase producers are also resistant to extra β-lactams and are not inhibited by current β-lactamase inhibitors (Jacoby, 2009). WHO and OIE classify β-lactams hydrolysed by ESBL and AmpC β-lactamases as critically important for both human and animal health (FAO/WHO/OIE, 2007).

The production and transfer of ESBL/AmpC β-lactamase determinants have contributed to the global infection control dilemma, and *E. coli* is among the six drug-resistant microbes to which new therapies are urgently needed (Shah *et al*., 2004; Da Silva and Mendonça, 2012).

Despite the prevalence of ESBL/AmpC β-lactamase producing *E. coli* in healthcare settings, these bacteria have emerged as causes of gastrointestinal infections acquired in the community even in the absence of selective pressure from AB use (Malik *et al*., 2005; Paterson, 2006). Furthermore, it has been reported that plasmids carrying CTX-M enzymes can transfer these determinants to other commensal Enterobacteriaceae, such as *Klebsiella pneumoniae*, or to pathogens like *Shigella* or *Salmonella* spp. (Woerther *et al*., 2011). Additionally, plasmid-mediated AmpC β-lactamase producers readily spread resistance to other bacteria both in hospital and community settings (Jacoby, 2009). ESBL/AmpC β-lactamase-bearing isolates are, therefore, significant in not only pathogenic bacteria but also commensals, which might be important gene reservoirs (Smet *et al*., 2003).

There has been evidence that food could be an important source of AB resistance genes either through consumption or cross-contamination (Witte, 2000; Depoorter *et al*., 2012; Ma *et al*., 2012). Transfer of resistance genes usually occurs through the consumption of food and either direct contact with food animals or other environmental mechanisms (WHO, 2011). Abuse of ABs in food animals has important implications for public health, as it promotes the development of resistant bacteria and resistance genes that can be passed on to humans. The transfer of AB resistance genes between bacteria from terrestrial animals, fish and humans can further take place in various environments, such as kitchens, barns and water sources (EFSA, 2010).

ESBL/AmpC β-lactamases have been increasingly reported among commensal Enterobacteriaceae from food-producing animals between the years 2002 and 2009 at a prevalence of 0.2–40.7% (Smet *et al*., 2010). Highly similar ESBL producing *E. coli* strains have been reported in both humans and retail chicken products (Manges and Johnson, 2012). Transfer of resistant strains either through direct or indirect contact by consumption of animal products or by contact with surface water or vegetables contaminated with broiler excreta has been reported (Witte, 2000; Depoorter *et al*., 2012; Ma *et al*., 2012).

However, little attention has been given to the transfer of resistance genes through water and vegetables, although evidence has shown that it might be an important pathway of gene transfer to human pathogenic and commensal strains (Witte, 2000; Cocconcelli *et al*., 2003; Mølbak *et al*., 2003; Toomey *et al*., 2009; Depoorter *et al*., 2012). Further studies based on molecular typing of bacterial clones and of resistance genes in vegetable production environments have been recommended in order to facilitate knowledge of the relative importance of these pathways in resistance transfer through vegetables (Witte, 2000; Depoorter *et al*., 2012).

Fresh produce are increasingly utilized in minimally processed forms, and mobile genetic elements present in contaminating flora might be transferred to bacteria in the human gut after consumption. This is especially of concern after ESBL-coding SHV and CTX-M-1 gene sequences of Enterobacteriaceae from retail lettuce isolates in a recent study showed 100% homology with the ESBL sequences from clinical isolates (Bhutani *et al*., 2012). A high prevalence of AB multi-resistant *E. coli* isolates was detected from two irrigation water sources in South Africa and from lettuce irrigated with water from one of the sources (Aijuka *et al*., 2014). Further study will facilitate a more accurate risk assessment concerning the spread of AB resistance, as well as the transferability of ESBL determinants in natural environments (Smet *et al*., 2010).

To our knowledge, there are no studies of ESBL/AmpC gene transfer between microorganisms from irrigation water to fresh produce. Such data are important to the understanding of the putative spread of mobile genetic elements through the human food chain by environmental sources and possible mitigation points. *Escherichia coli* strains obtained from irrigation water and lettuce were characterized for ESBL/AmpC β-lactamases and phylotypes. Molecular genotyping data were also used to test hypotheses regarding the possible transmission history of ESBL/AmpC β-lactamases from irrigation water to lettuce and between irrigation water from different ecological compartments.

## Results

A screening test using extended-spectrum cephalosporins (ceftazidime, cefotaxime, ceftriaxone and cefpodoxime) and aztreonam revealed the presence of ESBLs in 28 (65%) isolates. Confirmation by a double-disc synergy test confirmed only 13 (28.3%) of the isolates as ESBL positive (data not shown).

**Fig 1:**
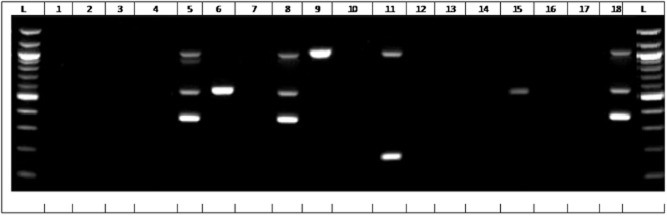
Illustrative multiplex PCR III for ACC (ACC-1 and ACC-2), FOX (FOX-1 to FOX-5), MOX (MOX-1, MOX-2, CMY-1, CMY-8 to CMY-11 and CMY-19), DHA (DHA-1 and DHA-2) and CIT (LAT-1 to LAT-3, BIL-1, CMY-2 to CMY-7, CMY-12 to CMY-18 and CMY-21 to CMY-23). Lanes L, DNA ladder; 1, RNAse free sterile water; 2, *E*. *coli* W1.8; 3, *E*. *coli* W1.9; 4, *E. coli* W 1.11; 5, *E. coli* L1; 6, *E. coli* W2.6; 7, *E. coli* W1.3; 8, *E. coli* W2.8; 9, *E*. *coli* W1.15; 10, *E. coli* W1.4; 11, *E*. *coli* L7; 12, *E. coli* W2.1; 13, *E. coli* W2.2; 14, *E. coli* W2.3; 15, *E. coli* W2.10; 16, *E. coli* W2.7; 17, *E. coli* W1.1; 18, *E. coli* W2.9; L, Quick-load, 100 bp DNA ladder (Biolabs New England). Expected amplicon sizes were 162 bp (FOX), 346 bp (ACC), 538 bp (CIT), 895 bp (MOX) and 997 bp (DHA).

Figure 1 presents an illustrative multiplex polymerase chain reaction (PCR) III for ACC (ACC-1 and ACC-2), FOX (FOX-1 to FOX-5), MOX (MOX-1, MOX-2, CMY-1, CMY-8 to CMY-11 and CMY-19), DHA (DHA-1 and DHA-2) and CIT (LAT-1 to LAT-3, BIL-1, CMY-2 to CMY-7, CMY-12 to CMY-18 and CMY-21 to CMY-23). The ESBL gene profiles differed significantly with the source (Table 1). ESBLs/AmpC β-lactamase genes were detected in 35 isolates (76%), with prevalence highest in lettuce (90% of isolates), followed by canal water (73%) and river water (64%) (Table 1). Plasmid-mediated AmpC β-lactamase genes were observed in 23 isolates (50%), ESBLs were observed in 27 (59%), and 14 isolates (30%) contained both an ESBL and a plasmid-mediated AmpC β-lactamase.

**Table 1:** Prevalence of extended-spectrum and AmpC β-lactamases in *E. coli* isolated from two irrigation water sources and lettuce

	Source		
ESBL variants	CW (*n =* 22)	RW (*n =* 14)	RL (*n =* 10)
TEM variants^a^	5 (1)	–	30 (3)
SHV variants^b^	–	7 (1)	0
CTX-M group 1^c^	18 (4)	14 (2)	10 (1)
CTX-M group 2^d^	–	7 (1)	–
CTX-M group 9^e^	–	7 (1)	–
CTX-M group 8/25^f^	55 (12)	36 (5)	80 (8)
ACC^g^	23 (5)	21 (3)	30 (3)
MOX^h^	14 (3)	–	30 (3)
CIT^i^	18 (4)	43 (6)	30 (3)
DHA^j^	23 (5)	43 (6)	40 (4)
FOX^k^	–	–	10 (1)
OXA-1^l^	–	–	–

a TEM variants including TEM-1 and TEM-2.

b SHV variants including SHV-1.

c CTX-M group 1 variants including CTX-M-1, CTX-M-3 and CTX-M-15.

d CTX-M group 2 variants including CTX-M-2.

e CTX-M group 9 variants including CTX-M-9 and CTX-M-14.

f CTX-M-8, CTX-M-25, CTX-M-26 and CTX-M-39 to CTX-M-41.

g ACC-1 and ACC-2.

h MOX-1, MOX-2, CMY-1, CMY-8 to CMY-11 and CMY-19.

i LAT-1 to LAT-3, BIL-1, CMY-2 to CMY-7, CMY-12 to CMY-18 and CMY-21 to CMY-23.

j DHA-1 and DHA-2.

k FOX-1 to FOX-5.

l OXA-1, OXA-4 and OXA-30.

ESBL profiles differed significantly with the source χ^2^ (6, *n =* 43) = 39.4%, *P* < 0.001.

–, not detected; CW, river from Mpumalanga province; RW, canal in North West province; RL, lettuce irrigated with water from RW; percentages are calculated as the number of strains with a given ESBL/AmpC β-lactamase profile divided by the total number of strains from the respective source; number of positive isolates in parentheses.

Resistance was detected for all of 11 tested enzyme groups. Major ESBL determinants were of the CTX-M type. CTX-M type ESBLs were found in 73% (16 isolates), 64% (9 isolates) and 90% (9 isolates) of the isolates from Loskop canal water, Skeerpoort river water and lettuce respectively. A majority of the CTX-M (25 isolates) were from group 8/25. Seven isolates also had CTX-M 2 type ESBLs. DHA (33% of isolates), CIT (28% isolates) and ACC (24% isolates) were the dominant isolated plasmid-mediated AmpC β-lactamases.

Twenty-two isolates (48%) contained between two and five ESBL/AmpC β-lactamase resistance genes. Six isolates carried three β-lactamases, six carried two β-lactamases, seven carried four β-lactamases and two carried five β-lactamase genes (Table 2; Fig. 1). Six (60%) of the isolates from lettuce were multi-resistant (Table 2). In 18 of the 22 isolates containing more than one ESBL/AmpC β-lactamase, there were one or more AmpC β-lactamases accompanied by ESBLs. The most common multi-resistance combinations among the isolates were the CTX-M Group 8/25 (17 isolates) combined with either the AmpC β-lactamase group CIT (13 isolates), DHA (12 isolates) or ACC (12 isolates) (Table 2; Fig. 1).

**Table 2:** Extended-spectrum and AmpC β-lactamase multi-resistant *E. coli* isolated from two irrigation water sources and lettuce

		Resistance profile^a^^b^									
Strain	Source^c^	TEM	SHV	CTX-M group 1	CTX-M group 2	CTX-M group 8/25	ACC	MOX	CIT	DHA	FOX
W2.3	RW		x		x	x					
W2.6	RW					x			x		
W2.8	RW					x	x		x	x	
W2.9	RW					x	x		x	x	
W2.10	RW			x					x		
W2.11	RW					x	x		x	x	
W2.14	RW						x		x	x	
LW2.1	RL	x					x	x	x	x	
LW2.2	RL					x	x	x	x		
LW2.3	RL			x		x				x	
LW2.4	RL	x				x			x	x	
LW2.7	RL					x				x	x
LW2.10	RL	x				x	x	x			
W1.1	CW			x		x					
W1.2	CW			x		x	x	x			
W1.7	CW						x		x	x	
W1.12	CW	x				x					
W1.13	CW					x	x	x	x		
W1.15	CW					x				x	
W1.16	CW			x		x	x		x	x	
W1.17	CW						x		x	x	
W1.18	CW			x		x					

a Variants explained in Table 1 footnote.

b CW, river from Mpumalanga province, RW, canal in North West province, RL, lettuce irrigated with water from RW.

c x means positive.

The phylotypes of *E. coli* differed significantly based on the source (Table 3). Strains from phylogenetic groups A (26%) and B1 (46%) were the most common, followed by phylogenetic group D (20%) and B2 (9%) (Table 3).

**Table 3:** Distribution of phylogenetic groups of *E. coli* strains from irrigation water and lettuce

	Source		
Phylogenetic group	MPUW (*n =* 22)	NWW (*n =* 14)	NWL (*n =* 10)
A	23 (5)	29 (4)	30 (3)
B1	46 (10)	43 (6)	50 (5)
B2	9 (2)	14 (2)	–
D	23 (5)	14 (2)	20 (2)

Phylotypes differ significantly with the source χ^2^ (6, *n =* 43) = 17, *P =* 0.09.

–, not detected; CW, river from Mpumalanga province; RW, canal in North West province; RL, lettuce irrigated with water from RW; percentages are calculated as the number of strains with a given ESBL/AmpC profile over the total number of strains from the respective source; number of isolates in parentheses.

**Fig 2:**
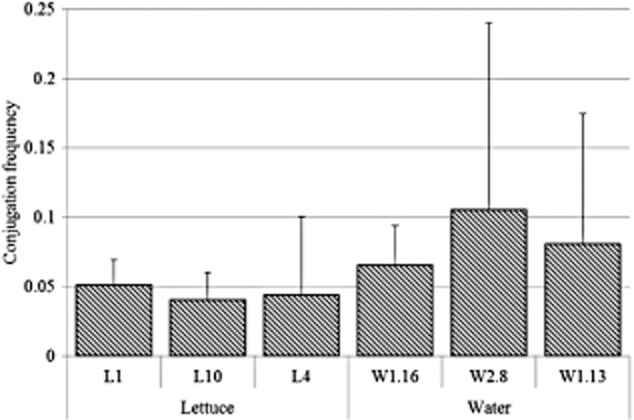
The frequency of conjugative ESBL/AmpC β-lactamase resistance gene transfer among *E. coli* from lettuce and irrigation water. Vertical bars represent standard deviations.

**Fig 3:**
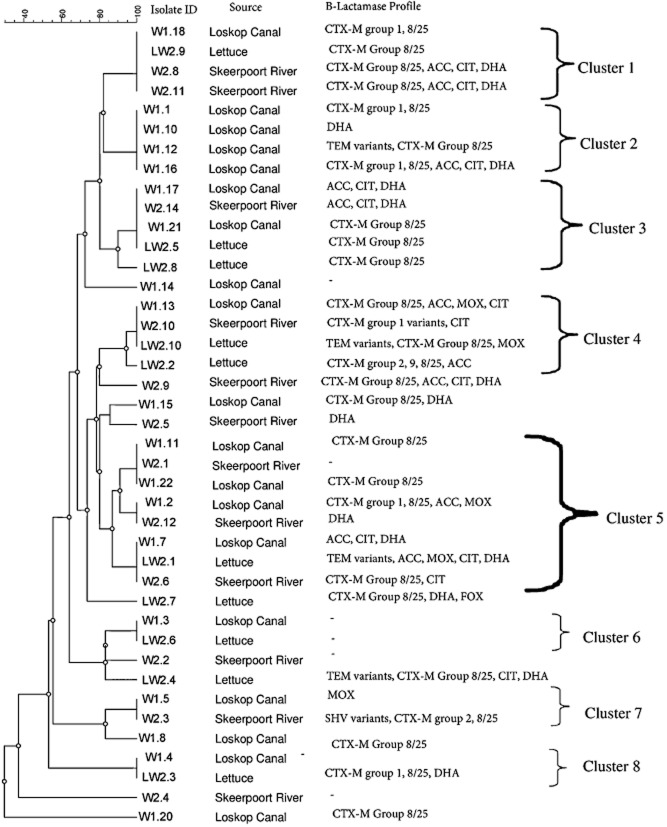
Dendrogram for REP-PCR fingerprints of *E. coli* isolates obtained from irrigation water and lettuce and their ESBL/AmpC β-lactamase resistance profiles^a^. Calculations were based on the Jaccard similarity coefficient using an unweighted pair group method with arithmetic average dendrogram type, 1.30% position tolerance and 2.00% optimization. ^a^Variants explained in Table 1 footnote. Clusters defined at ≥ 85% similarity.

Repetitive extragenic palindromic PCR (rep-PCR) fingerprinting enabled the study of *E. coli* strain inter-relatedness and evidence of potential transmission of ESBLs/AmpC β-lactamases in *E. coli* from irrigation water and lettuce (Fig. 2). There were eight clusters of isolates. Four clusters included lettuce and water from the source used to irrigate the lettuce (Fig. 2). Six of the clusters showed similarity between isolates from the two water sources (Fig. 2). Similar β-lactamases in isolates from lettuce and lettuce irrigation water in the same clusters included cluster 1 (CTX-M 8/25), cluster 4 (CIT) and cluster 5 (DHA and CTX-M 8/25) (Fig. 2). Lettuce from clusters 3 and 4 also had intra-cluster similarity in the ESBL CTX-M 8/25. All of the clusters except the first one showed several differences in β-lactamase profiles in strains from similar sources and also irrigation water and lettuce (Fig. 2). The conjugation efficiency of the isolates ranged from 3.5 × 10^−2^ to 1 × 10^−2^ ± 1.4 × 10^−1^ (Fig. 3). Water isolates (*P* < 0.05; μ = 8.4 × 10^−2^ ± 2 × 10^−2^; Pearson correlation *=* 0.96) had a significantly higher conjugation frequency (*P =* 0.04) than those from lettuce (μ = 4.5 × 10^−2^ ± 5.4 × 10^−3^; Pearson correlation *=* 0.26) (Fig. 3).

## Discussion

Lettuce, like most other fresh produce, does not undergo microbial inactivation or preservation treatment but undergoes only partial interventions like a chlorine wash. Due to this lack of intervention treatments, viable bacteria, whenever they are present, may persist along the food chain, and consumers could therefore be exposed to ESBL/AmpC β-lactamase-harbouring *E. coli*. The recent increase in fresh produce consumption may also enhance the possibility of human acquisition of bacteria producing ESBL/AmpC β-lactamases (Lynch *et al*., 2009). However, there are few studies exploring this possibility, even though evidence suggests a contribution of fresh produce bearing multi-resistant Enterobacteriaceae to resistance determinants in human commensal and pathogenic bacteria (Boehme *et al*., 2004).

Initial screening tests with extended-spectrum cephalosporins and aztreonam revealed the presence of ESBLs in 65%, which reduced to 28.3% when a double-disc synergy test was used for confirmation. The reduced prevalence in confirmatory tests is because bacteria producing AmpC β-lactamase show a positive ESBL screening test followed by a negative increased clavulanic acid-sensitivity confirmatory test. This effect has also been noted for some TEM mutants, OXA-type ESBLs and carbapenemases (Jacoby, 2009). Molecular typing also detected a higher prevalence (76%) of ESBL/AmpC β-lactamase-bearing *E. coli* than was detected by phenotypic tests. Therefore, the ‘gold standard’ for detection of bacteria harbouring plasmid-mediated ESBL/AmpC β-lactamases is molecular techniques (Pitout and Laupland, 2008; Falagas and Karageorgopoulos, 2009; Jacoby, 2009).

Table 4 compares the prevalence in ESBL/AmpC β-lactamases from the current study with those reported in literature from *E. coli* or other members of the Enterobacteriaceae family of food or food animal origin. This study reveals a high prevalence of ESBL/AmpC β-lactamases in lettuce and irrigation water when compared with other food and food animals. Comparison of AB resistances in microorganisms between studies serves as an indicator of prevalence, but it does not prove actual differences in prevalence due to differences in the types of samples, methods of strain isolation or approaches in testing for resistance (Smet *et al*., 2010; EFSA, 2011; Holvoet *et al*., 2013). There are few studies on ESBL/AmpC β-lactamases in fresh produce, and most reports are on food and food animals. The reported prevalence of ESBL in isolates from vegetables or fruits was found to be 2.3% (Saudi Arabia), 5% (Netherlands) and 49.9% (France), with the highest prevalence being 90% (Canada) (Table 4). The prevalence of ESBL-carrying *E. coli* in food-producing animals varies between the species, region and period studied (Table 4). In chicken, ESBL occurrence ranged from a low of 6.3% (USA) to a high of 92% (Netherlands), whereas the range was 3–13% in turkey (USA) (Table 4). In cattle and cattle products, the prevalence ranged between 0.5% (ground beef from USA) and 6.5% (cattle from 10 European Union countries). Low levels of resistance ranging from 0.7% to 6.8% were reported in pig products (pork chops from USA) (Table 4). The overall prevalence of 76% in ESBL/AmpC β-lactamase genes in *E. coli* in the present study and prevalence of 90% in lettuce are therefore high when viewed with respect to other reports.

**Table 4:** Prevalence of ESBL and AmpC β-lactamases in *E. coli* isolates from lettuce from this study compared with other foods and food animals

β-lactam/β-lactamase	Source	Prevalence	Country/region	Period	Reference
ESBL and/or AmpC	Lettuce	90	Republic of South Africa	2014	This study
β-lactam/β-lactamase	Chicken meat	6.7–14.1	USA	2002–2011	FDA (2013)
Inhibitor combination					
Ceftriaxone	Chicken	6.3–13.5	USA	2002–2008	Tadesse and colleagues (2012)
Cefotaxime	Chicken	8.5–26	8 EU countries	2009	EFSA (2011)
ESBL and/or AmpC	Chicken	80	Netherlands	2010	Dierikx and colleagues (2013)
ESBL and/or AmpC	Chicken meat	92	Sweden, imported from South America	2011	Börjesson and colleagues (2011)
ESBL and/or AmpC	Chicken meat	30–36	Denmark and UK, imported from South America	2009	EFSA (2011)
Ceftiofur	Chicken meat	21–34	Canada	2004	Li and colleagues (2007)
β-lactam/β-lactamase	Ground turkey	3–13	USA	2002–2011	FDA (2013)
Inhibitor combination					
β-lactam/β-lactamase	Ground beef	0.5–3.9	USA	2002–2011	FDA (2013)
Inhibitor combination					
Cefotaxime	Cattle	1.6–6.5	10 EU countries	2009	EFSA (2011)
β-lactam/β-lactamase	Pork chop	0.7–6.8	USA	2002–2011	FDA (2013)
Inhibitor combination					
Cefotaxime	Pig	2.3–3.8	EU	2009	EFSA (2011)
ESBLs^a^	Vegetables	5	Netherlands	2010	Reuland and colleagues (2011b)
ESBL^a^	Vegetables	2.3	Saudi Arabia	2011	Hassan and colleagues (2011)
Third-generation cephalosporins	Fruits and vegetables	49.9	France	2003–2004	Ruimy and colleagues (2010)
B-Lactams	Raw salad vegetables	> 90	Canada	2008	Bezanson and colleagues (2008)
CTX-M	Lettuce	90	Republic of South Africa	2014	This study
ACC	Lettuce	30	Republic of South Africa	2014	This study
CIT	Lettuce	30	Republic of South Africa	2014	This study
DHA	Lettuce	40	Republic of South Africa	2014	This study
CTX-M-14	Chicken	1.3	Spain	2003	Carattoli (2009)
CTX-M-9	Chicken	0.3	Spain	2003	Carattoli (2009)
TEM variants	Chicken	2–13	Belgium	2008	Smet and colleagues (2008)
CTX-M variants	Chicken	2–27	Belgium	2008	Smet and colleagues (2008)
CMY-2	Chicken	49	Belgium	2008	Smet and colleagues (2008)
CTX-M-9, CTX-M-14 and SHV-12	Chicken at slaughter	5	Spain	2003	EFSA (2011)
AmpC	Chicken	0.8–3.3	Various EU and Asian countries	–	EFSA (2011)
blaCTXM-14	Cattle	66.7	France	2012	Dahmen and colleagues (2013)
CTX-M-2	Cattle	1.5	Japan	2000–2001	Carattoli (2009)
AmpC	Cattle	2.4–23	Canada, Taiwan, Mexico	–	EFSA (2011)
blaTEM	Duck	56.7	China	2006	Ma and colleagues (2012)
blaSHV	Duck	4.1	China	2006	Ma and colleagues (2012)
blaCTX-M	Duck	87.8	China	2006	Ma and colleagues (2012)
blaCMY	Duck	7.5	China	2006	Ma and colleagues (2012)
blaDHA	Duck	80	China	2006	Ma and colleagues (2012)

a Enterobacteriaceae.

–, not indicated; EU, European Union.

The prevalence of resistance was highest in lettuce, followed by the canal water and the river water. Holvoet and colleagues (2013) similarly reported a higher prevalence in resistance to ABs in *E. coli* isolated from lettuce (22%) than those from soil (8.8%) or irrigation water samples (7.5%).

The major ESBL determinants detected were of the CTX-M type (Table 1), and the prevalence of CTX-M type ESBLs genes in lettuce was 90%. The CTX-M type ESBL is of increasing concern globally. The CTX-M, TEM and SHV families have been reported as the predominant ESBLs, whereas CMY has been reported as the predominant AmpC β-lactamase in isolates from foods. Whereas TEM- and SHV-type ESBLs predominate hospital-acquired infections worldwide, the CTX-M family consists of 70% of ESBL in *E. coli* from community-onset infections (Paterson, 2006). Globally, 79% of ESBLs harboured by *E. coli* isolates of animal and animal food origin belong to CTX-M-type variants, especially CTX-M-14, followed by CTX-M-1. However, the prevalence of certain variants is influenced by animal species (Torres and Zarazaga, 2007). The CTX-M β-lactamases are an increasing and important group because they mediate high-level resistance not only to penicillins and narrow-spectrum first- and second-generation cephalosporins but also to third-generation cephalosporins, as well as variable levels of resistance to the fourth-generation cephalosporins (Stürenburg and Mack, 2007; Li *et al*., 2007). A high percentage of resistance to *bla*RAHN-1, which is closely related to *bla*CTX-M, was detected in all of the 51 ESBL phenotypic-positive Gram-negative bacteria isolated from fruits and vegetables (Bezanson *et al*., 2008). Enterobacteriaceae harbouring CTX-M genes were recently reported in spinach, parsnips, bean sprouts and radishes (Raphael *et al*., 2011; Reuland *et al*., 2011a). CTX-M-15-producing *E. coli* O104:H4 was implicated in an outbreak associated with contaminated sprouts (EFSA, 2011). In chicken, CTX-M resistance ranged from 0.3–1.3% (Spain) to 27% (Belgium). CTX-M ranged from 1.5% in cattle (Japan) to 66.7% (France). The prevalence of CTX-M was 87.8% in a duck farm in China (Table 4).

Compared with other studies, the AmpC β-lactamase prevalence in this study was moderate. A high occurrence of DHA (80%) was reported in a duck farm in China (Table 4). The incidence of CMY-2, which belongs to the CIT group, was high (49%) in broiler farms (Belgium), whereas a low incidence (7.5%) of CMY was reported in a duck farm (China). However, even the low occurrence of Amp C β-lactamase in *E. coli* is a matter of concern because high-level expression of Amp C β-lactamases has been identified in clinical specimens. Increased production of chromosomal AmpC β-lactamases associated with the possession of plasmid-mediated AmpC β-lactamases is a major threat (Jacoby, 2009). A recent increase in CMY-2 producers, especially in the USA, has been associated with use of ceftiofur and possibly with efficient horizontal transmission of its encoding plasmids (Carattoli, 2009).

We detected multi-resistance in 48% of the isolates that contained between two and five ESBL/AmpC β-lactamase resistance genes. Resistance to as many as eight β-lactamases has been reported especially in hospital-acquired pathogens (Moland *et al*., 2007). In 18 of the 22 multi-resistant isolates, there were one or more AmpC β-lactamase accompanied by ESBLs. AmpC β-lactamase plasmids often harbour multiple resistance genes, including the β-lactamase gene varieties TEM-1, CTX-M and SHV also reported in this study (Jacoby, 2009). Furthermore, CMY- or CTX-M-encoding plasmids often contain multiple resistance determinants and have also been associated with transposons and integrons (Li *et al*., 2007).

The phylotypes of *E. coli* from this study differed significantly with the source (Table 3). The different patterns of distribution of the phylogenetic groups among the three sources can be attributed to factors including geographic/climatic conditions and host genetic factors on commensal flora (Duriez *et al*., 2001). Strains from phylogenetic groups A (26%) and B1 (46%) were the most common, followed by phylogenetic group D (20%) and B2 (9%) (Table 3). Similar profiles to those of *E. coli* in the current study have also been reported in human strains from different geographical regions. Group A (40%) and B1 (34%) strains were previously reported as the most prevalent. Group D and B2 followed at a prevalence of 15% and 9–11% respectively (Goullet and Picard, 1986; Duriez *et al*., 2001). This profile is unique to that of human isolates. For instance, occurrence of the rare group B2 phylotype in animal isolates is reported at 1.6% (Goullet and Picard, 1986). This profile suggests a possible link between contamination in the production environment (water and field lettuce), with *E. coli* harbouring ESBL/AmpC β-lactamase determinants and human sources. In this study, all of the strains from phylogroups B2 (4/4) and 78% (7/9) of those from D were ESBL/AmpC β-lactamase positive. In contrast, all except one of the susceptible *E. coli* in this study belonged to phylogenetic groups A and B1. A majority of *E. coli* from group B2 (75%) and 33% of isolates from group D were reported by Aijuka and colleagues (2014) to harbour the virulence genes *eae* and *stx*1/*stx*2. The strains from phylogroups B2 and D may, therefore, be significant health threats given that a majority of their members are both ESBL/AmpC β-lactamase positive and also contain virulence genes.

Repetitive extragenic palindromic PCR enabled the study of *E. coli* strain inter-relatedness and evidence of a history of transmission of ESBLs/AmpC β-lactamase determinants between the water sources and lettuce (Fig. 2). The eight clusters of isolates included similar β-lactamase profiles in isolates from lettuce and lettuce irrigation water. Such a high degree of genetic relatedness between strains is an indicator of common ancestry, which outlines a pathway of transmission (Olsen *et al*., 1993; Salamon *et al*., 2000; Weigel *et al*., 2007). This evidence was further supported by the high conjugation efficiency of the *E. coli* isolates (Fig. 3). Isolates from water were more adapted to gene transfer than those from lettuce. Furthermore, when total variation in conjugation efficiency was considered, isolates from water explained the largest proportion of the variation (Pearson correlation coefficient of 0.96).

Several differences in β-lactamase profiles were also noted in strains from similar sources, and similar genes in *E. coli* from irrigation water and lettuce were present within all clusters except the first. Such diverse patterns indicate horizontal transfer rather than the pandemic spread of single strains. Differences in resistance profiles among strains from tight clusters can be explained because in many cases, resistant bacteria adapt quite well and more stably maintain their own AB resistance genes when in the same ecosystem as other resistant strains (Boehme *et al*., 2004). For the two irrigation water sources, which are approximately 246 km distant apart, the segregation of *E. coli* genotypes and ESBL/AmpC β-lactamase profiles, as indicated by six similar clusters, suggests similarities between the *E. coli* transmissions despite spatial distance. This similarity is further supported by cluster analysis, which revealed two main AB multi-resistance clusters (Fig. S1). A study between multi-site farms detected 25% of tight salmonella clusters from different sites, indicating transmissions between sites (Weigel *et al*., 2007). The emergence of resistance even in particular regions has a global significance through the spread of resistance worldwide, which is associated with increase in morbidity, mortality and healthcare costs (Sundsfjord *et al*., 2004).

The role of irrigation water and the soil production environment in the spread of bacteria resistant to various ABs is emerging. AB-resistant bacteria prevalence from 72% to 100% for faecal coliforms and 87% for non-faecal coliforms has been reported in domestic sewage, drinking water, rivers and lakes (Sayah *et al*., 2005). Resistance was found in animal faecal samples to all 12 of the ABs tested, whereas river water and human septage samples showed resistance to one and three ABs respectively (Sayah *et al*., 2005). Among the β-lactamases, Sayah and colleagues (2005) reported resistance to cephalothin in all samples. Similar resistance profiles in *E. coli* from animal faecal and farm environment samples among different animal species suggested common sources of the resistant bacteria (Sayah *et al*., 2005).

In conclusion, we report a high prevalence of ESBL and a moderate prevalence AmpC β-lactamase determinants in *E. coli* from lettuce and irrigation water. Genetic similarities in the resistant isolates from irrigation water and lettuce indicate that irrigation water likely contributes to ESBL/AmpC β-lactamases in both commensal and pathogenic bacteria found in lettuce. Both the transfer of mobile genetic elements and the direct transfer of strains from irrigation water are suggested. Commensal *E. coli* may contribute to the maintenance and dissemination of ESBL/AmpC β-lactamase determinants. The close similarity in the phylogenetic profiles of the *E. coli* isolates from lettuce and water compared with those of humans links human contamination to *E. coli* harbouring ESBL/AmpC β-lactamase determinants in lettuce production environments, especially irrigation water. The strains from phylogroups B2 and D may form significant health threats given that a majority contain both ESBL/AmpC β-lactamase as well as virulence genes. ESBL/AmpC β-lactamase genes are transferrable from *E. coli* in irrigation water to bacteria in lettuce. *Escherichia coli* from lettuce have potential to be maintenance and transfer agents of ESBL/AmpC β-lactamase genes to intra- and extra-intestinal pathogens. The lack of reports describing transmission of important β-lactamase determinants to microbial contaminants in vegetables from the production environment might lead to an underestimation of this route of transmission when compared with animal foods. This route of transmission raises serious concerns, given that ESBL/AmpC β-lactamases hydrolyse ABs that are critically important for both human and animal health. Further quantitative risk analysis is needed, taking into consideration growth during transport, retail handling and consumption, as well as dose response. This further analysis will provide information about the actual risk to humans incurred from the consumption of such lettuce.

## Experimental procedures

### *E. coli* isolates

*Escherichia coli* strains were previously isolated and identified over 10 months in the summer, fall, winter and spring of 2011 in South Africa. Water samples were obtained from an irrigation canal (CW; *n =* 22) in Mpumalanga province, a river in North West province (RW; *n =* 12) and lettuce irrigated with water from this river (RL; *n =* 10) (Aijuka *et al*., 2014). The two water sources are approximately 246 km apart. Seven isolates harboured either single or combinations of the virulence genes *eae*, *stx*1/*stx*2. Thirty seven of the isolates were either resistant or intermediate resistant to two or more ABs tested, including amikacin, gentamicin, nalidixic acid, norfloxacin, neomycin, nitrofurantoin, ampicillin, oxytetracycline, amoxicillin, neomycin and cephalothin (Aijuka *et al*., 2014).

### β-lactamase screening of *E. coli* isolates

*Escherichia coli* isolates were screened for ESBL using a disc diffusion test with expanded-spectrum cephalosporins (which are hydrolysed by all TEM, SHV and CTX-M types of ESBLs) and aztreonam on Mueller-Hinton II agar (Pitout *et al*., 1997; Pitout and Laupland, 2008; Smet *et al*., 2008; Falagas and Karageorgopoulos, 2009). The cephalosporins used were ceftazidime (30 μg), cefotaxime (30 μg), ceftriaxone (30 μg) and cefpodoxime (10 μg) (Bio-Rad, Laboratories, Hercules CA). Extended-spectrum β-lactamase phenotypic production was confirmed using the modified double-disc diffusion method or the combined-disc method (Stürenburg and Mack, 2007). Cefotaxime + clavulanic acid (30 μg + 10 μg) and ceftazidime + clavulanic acid (30 μg + 10 μg) discs were used (Bio-Rad Laboratories). Extended-spectrum β-lactamase was positive when the zone diameters given by the discs with clavulanate were ≥ 5 mm larger than those without the inhibitor for at least one of the combinations. *Escherichia coli* ATCC 25922 (ESBL negative), *E. coli* ATCC 35218 (ESBL positive control), *K. pneumonia* ATCC 700603 (ESBL positive) and *Pseudomonas aeruginosa* ATCC 27853 (ESBL negative) strains were used as control strains for test performance.

### Molecular profiling of *E. coli* isolates

#### β-lactamase genes

DNA was extracted using a ZR Fungal/Bacterial DNA MiniPrep kit (Zymo Research, Irvine, CA). Three multiplex PCRs and one single PCR (Dallenne *et al*., 2010) were used to distinguish between four enzyme groups responsible for ESBL/AmpC β-lactamases. These included (i) multiplex I for TEM (variants including TEM-1 and TEM-2), SHV (variants including SHV-1) and OXA-1-like (OXA-1; OXA-1, OXA-4 and OXA-30); (ii) multiplex II for CTX-M group 1 (including CTX-M-1, CTX-M-3 and CTX-M-15), group 2 (including CTX-M-2) and group 9 (CTX-M-9 and CTX-M-14); (iii) CTX-M group 8/25 (CTX-M-8, CTX-M-25, CTX-M-26 and CTX-M-39 to CTX-M-41); and (iv) multiplex III for ACC (ACC-1 and ACC-2), FOX (FOX-1 to FOX-5), MOX (MOX-1, MOX-2, CMY-1, CMY-8 to CMY-11 and CMY-19), DHA (DHA-1 and DHA-2) and CIT (LAT-1 to LAT-3, BIL-1, CMY-2 to CMY-7, CMY-12 to CMY-18 and CMY-21 to CMY-23).

The 20 μl PCR mixture contained DNA (2 μl), 2 × HotStarTaq Plus Master Mix (Qiagen) (containing HotStarTaq Plus DNA polymerase, PCR buffer with 3 mM MgCl2, and 400 μM of each dNTP), 2 μl Q-solution, 2 μl CoralLoad concentrate, and a variable concentration of specific group primers as reported by Dallenne and colleagues (2010) with modifications. Polymerase chain reaction involved initial denaturation at 95°C for 5 min; 30 cycles of 94°C for 40 s, 60°C for 40 s and 72°C for 1 min; and a final elongation step at 72°C for 10 min (MiniOpticon Real-Time PCR System; Invitrogen). Amplicons were visualized after running at 120 V for 1 h on a 1.6% agarose gel containing 10 000X SYBR Safe DNA stain concentrate (Invitrogen) diluted 1:10 000 in agarose gel buffer. A 1 Kb Plus DNA Ladder (Invitrogen) or 100 bp DNA ladder (Biolabs New England) was used as a size marker.

Bidirectional sequencing of purified PCR products from selected isolates per positive ESBL/AmpC β-lactamase group was performed after simplex PCR in similar reaction conditions to those outlined above. Sequence analysis was performed at the Forestry and Agricultural Biotechnology Institute of the University of Pretoria. The gene sequences were analysed with the software FinchTV version 1.4.0 (Geospiza) and aligned using BioEdit (Hall, 1999). Comparison with available databases was done using the National Center for Biotechnology Information database matching (http://blast.ncbi.nlm.nih.gov/Blast.cgi). This acted as a confirmatory control of positive ESBL/AmpC β-lactamase gene groups from the PCR.

### Phylotyping and clonal grouping of *E. coli* strains

The phylogenetic group distribution of the isolates was typed to further compare and differentiate the strains as either commensal or potentially pathogenic strains. The *E. coli* strains were allocated to either phylogenetic groups A, B1, B2 or D using triplex PCR, targeting chuA, yjaA and tspE4C2 genes (Grasselli *et al*., 2008; Kluytmans *et al*., 2013). Modified rep-PCR, as outlined by Mohapatra and colleagues (2007), was used to evaluate the similarity between isolates from different sources. The 20 μl PCR mixture contained DNA (1 μl), 2 × master mix (Qiagen), 0.35 μM of (GTG)5 primer and 4% DMSO (Sigma-Aldrich, St Louis). Polymerase chain reaction involved initial denaturation at 95°C for 5 min; 35 cycles of 95°C for 30 s, 40°C for 60 s and 65°C for 3 min; and a final elongation step at 65°C for 8 min.

### In vitro conjugation

Transferability of β-lactamase resistance was measured by filter mating, as previously described (Woodall, 2003), with modifications. The frequency of β-lactamase gene transfer was studied using six multi-resistant donor strains (with four or five β-lactamase genes) consisting of three strains from water and three from lettuce. Conjugation experiments were performed by mating on sterile 0.45 μM nitrocellulose filter membranes (Merck Millipore). Recipient strains were selected based on ESBL/AmpC β-lactamase susceptibility and micro-dilution susceptibility tests targeting ampicillin, amoxicillin and tetracycline. The selected recipient was ESBL/AmpC β-lactamase susceptible and ampicillin resistant (at 32 μg ml^−1^). Conjugation frequency was calculated as the number of transconjugants divided by the total number of *E. coli* counted on Luria–Bertani agar plates. Colonies grown overnight from the highest dilution were plated on CHROMagar ESBL (CHROMagar Orientation base and CHROMagar ESBL supplement), and the plates were incubated at 37°C for 24 h. Typical dark pink to reddish colonies were regarded as ESBL producers. Experiments were conducted in triplicate.

### Data analysis

Hierarchical cluster analysis was performed on multi-resistance genetic profiles using xlstat version 2014.4.06. Repetitive extragenic palindromic PCR fingerprints were analysed using GelCompar II version 5.10 (Applied Maths, Sint-Martens-Latem, Belgium) software. The similarity among digitized profiles was calculated using the Pearson correlation, and an average-linkage dendrogram (using the unweighted pair group method with arithmetic averages) was derived from the profiles. Linking of isolates in tight clusters (similarity ≥ 85%) from different sources was regarded as evidence for transmission (Weigel *et al*., 2007).

A test for the association of ESBL/AmpC resistance profiles and phylogroups with the source was conducted by using 3 × 4 contingency tables, with two-tailed probabilities calculated using a chi-square test (alpha = 0.05). The rows included canal water (CW), river water (RW) and lettuce (RL), and the columns included ESBL group 1, ESBL group 2, ESBL group 3 and ESBL group 4 for β-lactamases, or A, B1, B2 and B2 for phylogroups.

Variability in conjugation frequency was modelled using the lognormal probability distribution in an Excel (Microsoft, Redmond, WA) spreadsheet add-in programme, @Risk (version 4.0, Palisade, Newfield, NY). Conjugation frequencies of *E. coli* from both from water and lettuce were treated as outputs, and the model was simulated to 10 000 iterations. The Spearman rank correlation between the conjugation frequencies from water and lettuce *E. coli* was calculated. One-way analysis of variance was conducted to examine the difference in conjugation frequency between *E. coli* from irrigation water and those from lettuce.

## Conflict of interest

None declared.

## Supporting Information

**Fig. S1.** Dendrogram representing cluster analysis of multi-resistant gene profiles among *E. coli* fingerprints of isolates obtained from irrigation water and lettuce. Agglomerative hierarchical similarity clustering was the analysis performed using an unweighted pair group average.

## References

[b1001] Aijuka M, Charimba G, Hugo CJ, Buys EM (2014). Characterization of bacterial pathogens in rural and urban irrigation water. J Water Health.

[b1] Bezanson GS, MacInnis R, Potter G, Hughes T (2008). Presence and potential for horizontal transfer of antibiotic resistance in oxidase-positive bacteria populating raw salad vegetables. Int J Food Microbiol.

[b2] Bhutani N, Talreja D, Walia S, Muraleedharan C, Kumar A, Rana SW (2012). http://www.abstractsonline.com/Plan/ViewAbstract.aspx?sKey=4ca432d4-62da-4f34-a6b9-fbfe2e656547&cKey=8fdcb57a-a59e-48d4-b806-5bb05c4cecc9&mKey={6B114A1D-85A4-4054-A83B-04D8B9B8749F}]Ent.

[b3] Boehme S, Werner G, Klare I, Reissbrodt R, Witte W (2004). Occurrence of antibiotic resistant enterobacteria in agricultural foodstuffs. Mol Nutr Food Res.

[b4] Börjesson S, Egervärn M, Finn M, Tillander I, Wiberg C, Englund S (2011). High prevalence of ESBL-producing *Escherichia coli* in chicken meat imported into Sweden. Clin Microbiol Infect.

[b5] Bush K, Jacoby GA (2010). Updated functional classification of beta-lactamases. Antimicrob Agents Chemother.

[b6] Carattoli A (2009). Animal reservoirs for extended spectrum β-lactamase producers. Clin Microbiol Infect.

[b7] Cocconcelli PS, Cattivelli D, Gazzola S (2003). Gene transfer of vancomycin and tetracycline resistances among *Enterococcus faecalis* during cheese and sausage fermentations. Int J Food Microbiol.

[b8] Da Silva GJ, Mendonça N (2012). Association between antimicrobial resistance and virulence in *Escherichia coli*. Virulence.

[b9] Dahmen S, Metayer V, Gay E, Madec J-Y, Haenni M (2013). Characterization of extended-spectrum beta-lactamase (ESBL)-carrying plasmids and clones of *Enterobacteriaceae* causing cattle mastitis in France. Vet Microbiol.

[b10] Dallenne C, Da Costa A, Decré D, Favier C, Arlet G (2010). Development of a set of multiplex PCR assays for the detection of genes encoding important beta-lactamases in Enterobacteriaceae. J Antimicrob Chemother.

[b11] Depoorter P, Persoons D, Uyttendaele M, Butaye P, De Zutter L, Dierick K (2012). Assessment of human exposure to 3rd generation cephalosporin resistant *E. coli* (CREC) through consumption of broiler meat in Belgium. Int J Food Microbiol.

[b12] Dierikx C, van der Goot J, Fabri T, van Essen-Zandbergen A, Smith H, Mevius D (2013). Extended spectrum beta-lactamase- and AmpC-beta-lactamase-producing *Escherichia coli* in Dutch broilers and broiler farmers. J Antimicrob Chemother.

[b13] Duriez P, Clermont O, Bonacorsi S, Bingen E, Chaventre A, Elion J (2001). Commensal *Escherichia coli* isolates are phylogenetically distributed among geographically distinct human populations. Microbiology.

[b14] EFSA (2010). The community summary report on antimicrobial resistance in zoonotic agents from animals and food in the European Union in 2004–2007. EFSA J.

[b15] EFSA (2011). Scientific opinion on the public health risks of bacterial strains producing extended-spectrum beta-lactamases in food and food-producing animals. EFSA J.

[b16] Falagas ME, Karageorgopoulos DE (2009). Extended-spectrum beta-lactamase-producing organisms. J Hosp Infect.

[b17] FAO/WHO/OIE (2007). http://www.who.int/foodborne_disease/resources/Report_CIA_Meeting.pdf.

[b18] FDA (2013). http://www.fda.gov/downloads/AnimalVeterinary/SafetyHealth/AntimicrobialResistance/NationalAntimicrobialResistanceMonitoringSystem/UCM334896.pdf.

[b19] Goullet P, Picard B (1986). Comparative esterase electrophoretic polymorphism of *Escherichia coli* isolates obtained from animal and human sources. J Gen Microbiol.

[b20] Grasselli E, François P, Gutacker M, Gettler B, Benagli C, Convert M (2008). Evidence of horizontal gene transfer between human and animal commensal *Escherichia coli* strains identified by microarray. FEMS Immunol Med Microbiol.

[b21] Hall TA (1999). BioEdit: a user-friendly biological sequence alignment editor and analysis program for Windows 95/98/NT. Nucleic Acids Symp Ser.

[b22] Hassan S, Altalhi A, Gherbawy Y, El-Deeb B (2011). Bacterial load of fresh vegetables and their resistance to the currently used antibiotics in Saudi Arabia. Foodborne Pathog Dis.

[b23] Holvoet K, Sampers I, Callens B, Dewulf J, Uyttendaele M (2013). Moderate prevalence of antimicrobial resistance in *Escherichia coli* isolates from lettuce, irrigation water, and soil. Appl Environ Microbiol.

[b24] Jacoby GA (2009). AmpC b-Lactamases. Clin Microbiol Rev.

[b25] Kluytmans JA, Overdevest IT, Willemsen I, Kluytmans-van den Bergh MF, van der Zwaluw K, Heck M (2013). Extended-spectrum β-Lactamase-producing *Escherichia coli* from retail chicken meat and humans: comparison of strains, plasmids, resistance genes, and virulence factors. Clin Infect Dis.

[b26] Li XZ, Mehrotra M, Ghimire S, Adewoye L (2007). Beta-lactam resistance and beta-lactamases in bacteria of animal origin. Vet Microbiol.

[b27] Lynch MF, Tauxe RV, Hedberg CW (2009). The growing burden of foodborne outbreaks due to contaminated fresh produce: risks and opportunities. Epidemiol Infect.

[b28] Ma J, Liu JH, Lv L, Zong Z, Sun Y, Zheng H (2012). Characterization of extended-spectrum β-lactamase genes found among *Escherichia coli* isolates from duck and environmental samples obtained on a duck farm. Appl Environ Microbiol.

[b29] Malik R, Ivan J, Javorsky P, Pristas P (2005). Seasonal dynamics of antibiotic-resistant Enterobacteriaceae in the gastrointestinal tract of domestic sheep. Folia Microbiol.

[b30] Manges AR, Johnson JR (2012). Food-borne origins of *Escherichia coli* causing extraintestinal infections. Clin Infect Dis.

[b31] Mohapatra BR, Broersma K, Mazumder A (2007). Comparison of five rep-PCR genomic fingerprinting methods for differentiation of fecal *Escherichia coli* from humans, poultry and wild birds. FEMS Microbiol Lett.

[b32] Moland ES, Hong SG, Thomson KS, Larone DH, Hanson ND (2007). A *Klebsiella pneumoniae* isolate producing at least eight different beta-lactamases including an AmpC and KPC beta-lactamase. Antimicrob Agents Chemother.

[b33] Mølbak L, Licht TR, Kvist T, Kroer N, Andersen SR (2003). Plasmid transfer from *Pseudomonas putida* to the indigenous bacteria on alfalfa sprouts: characterization, direct quantification, and in situ location of transconjugant cells. Appl Environ Microbiol.

[b34] Moubareck C, Lecso M, Pinloche E, Butel MJ, Doucet-Populaire F (2007). Inhibitory impact of bifidobacteria on the transfer of beta-lactam resistance among Enterobacteriaceae in the gnotobiotic mouse digestive tract. Appl Environ Microbiol.

[b35] Olsen JE, Brown DJ, Skov MM, Christensen JP (1993). Bacterial typing methods suitable for epidemiological analysis, applications in investigations of salmonellosis among livestock. Vet Q.

[b36] Paterson DL (2006). Resistance in gram-negative bacteria: *Enterobacteriaceae*. Am J Med.

[b37] Paterson DL, Bonomo RA (2005). Extended-spectrum beta-lactamases: a clinical update. Clin Microbiol Rev.

[b38] Pitout JD, Laupland KB (2008). Extended-spectrum beta-lactamase-producing *Enterobacteriaceae*: an emerging public-health concern. Lancet Infect Dis.

[b39] Pitout JDD, Sanders CC, Sanders WE (1997). Antimicrobial resistance with focus on beta-lactam resistance in gram-negative bacilli. Am J Med.

[b40] Raphael E, Wong LK, Riley LW (2011). Extended-spectrum Beta-lactamase gene sequences in Gram-negative saprophytes on retail organic and nonorganic spinach. Appl Environ Microbiol.

[b41] Reuland EA, Al Naiemi N, Rijnsburger MC, Savelkoul PH, Vandenbroucke-Grauls CM (2011a). Prevalence of ESBL-producing *Enterobacteriaceae* (ESBL-E) in raw vegetables. Clin Microbiol Infect.

[b42] Reuland EA, Al Naiemi N, Rijnsburger MC, Savelkoul PH, Vandenbroucke-Grauls CM (2011b). Prevalence of ESBL-producing Enterobacteriaceae (ESBL-E) in raw vegetables. Ned Tijdschr Med Microbiol.

[b43] Ruimy R, Brisabois A, Bernede C, Skurnik D, Barnat S, Arlet G (2010). Organic and conventional fruits and vegetables contain equivalent counts of Gram-negative bacteria expressing resistance to antibacterial agents. Environ Microbiol.

[b44] Salamon H, Behr MA, Rhee JT, Small PM (2000). Genetic distances for the study of infectious disease epidemiology. Am J Epidemiol.

[b45] Sayah RS, Kaneene JB, Johnson Y, Miller R (2005). Patterns of antimicrobial resistance observed in *Escherichia coli* isolates obtained from domestic- and wild-animal fecal samples, human septage, and surface water. Appl Environ Microbiol.

[b46] Shah AA, Hasan H, Ahmed S, Hameed A (2004). Extended spectrum β-lactamases (ESβLs): characterization, epidemiology and detection. Crit Rev Microbiol.

[b47] Smet A, Rasschaert G, Martel A, Persoons D, Dewulf J, Butaye P (2003). Extended-spectrum beta-lactamases: implications for the clinical microbiology laboratory, therapy, and infection control. J Infect.

[b48] Smet A, Martel A, Persoons D, Dewulf J, Heyndrickx M, Catry B (2008). Diversity of extended-spectrum beta-lactamases and class C beta-lactamases among cloacal *Escherichia coli* isolates in Belgian broiler farms. Antimicrob Agents Chemother.

[b49] Smet A, Martel A, Persoons D, Dewulf J, Heyndrickx M, Herman L (2010). Broad-spectrum β-lactamases among Enterobacteriaceae of animal origin: molecular aspects, mobility and impact on public health. FEMS Microbiol Rev.

[b50] Smith JL, Fratamico PM, Gunther NW (2007). Extraintestinal pathogenic *Escherichia coli*. Foodborne Pathog Dis.

[b51] Stürenburg E, Mack D (2003). Extended-spectrum β-lactamases: implications for the clinical microbiology laboratory, therapy, and infection control. J Infect.

[b1002] Sundsfjord A, Simonsen GS, Haldorsen BC, Haaheim SO, Hjelmevoll SO, Littauer P, Dahl KH (2004). Genetic methods for detection of antimicrobial resistance. APMIS.

[b52] Tadesse DA, Zhao S, Tong E, Ayers S, Singh A, Bartholomew MJ, McDermott PF (2012). Antimicrobial drug resistance in *Escherichia coli* from humans and food animals, United States, 1950–2002. Emerg Infect Dis.

[b53] Toomey N, Monaghan A, Fanning S, Bolton D (2009). Transfer of antibiotic resistance marker genes between lactic acid bacteria in model rumen and plant environments. Appl Environ Microbiol.

[b54] Torres C, Zarazaga M (2007). BLEE en animales y su importancia en la transmision a humanos. Enferm Infecc Microbiol Clin.

[b55] Weigel RM, Nucera D, Qiao B, Teferedegne B, Suh DK, Barber DA (2007). Testing an ecological model for transmission of *Salmonella enterica* in swine production ecosystems using genotyping data. Prev Vet Med.

[b56] WHO (2011). http://www.euro.who.int/__data/assets/pdf_file/0005/136454/e94889.pdf.

[b57] Wiegand I, Geiss HK, Mack D, Sturenburg E, Seifert H (2007). Detection of extended spectrum β-lactamases among *Enterobacteriaceae* by use of semi-automated microbiology systems and manual detection procedures. J Clin Microbiol.

[b58] Witte W (2000). Ecological impact of antibiotic use in animals on different complex microflora: environment. Int J Antimicrob Agents.

[b59] Woerther PL, Angebault C, Jacquier H, Hugede HC, Janssens AC, Sayadi S (2011). Massive increase, spread, and exchange of extended spectrum β-lactamase-encoding genes among intestinal Enterobacteriaceae in hospitalized children with severe acute malnutrition in Niger. Clin Infect Dis.

[b60] Woodall CA, Preston A, Casali N (2003). DNA transfer by conjugation. E. coli Plasmid Vectors: Methods and Applications.

