# Supplementary figures and images for: Pathogenic and commensal Escherichia coli from irrigation water show potential in transmission of extended spectrum and AmpC β-lactamases determinants to isolates from lettuce
Source: Microb Biotechnol. 2014 Dec 9;8(3):462–73. doi: 10.1111/1751-7915.12234 (PMC4408178; doi:10.1111/1751-7915.12234)

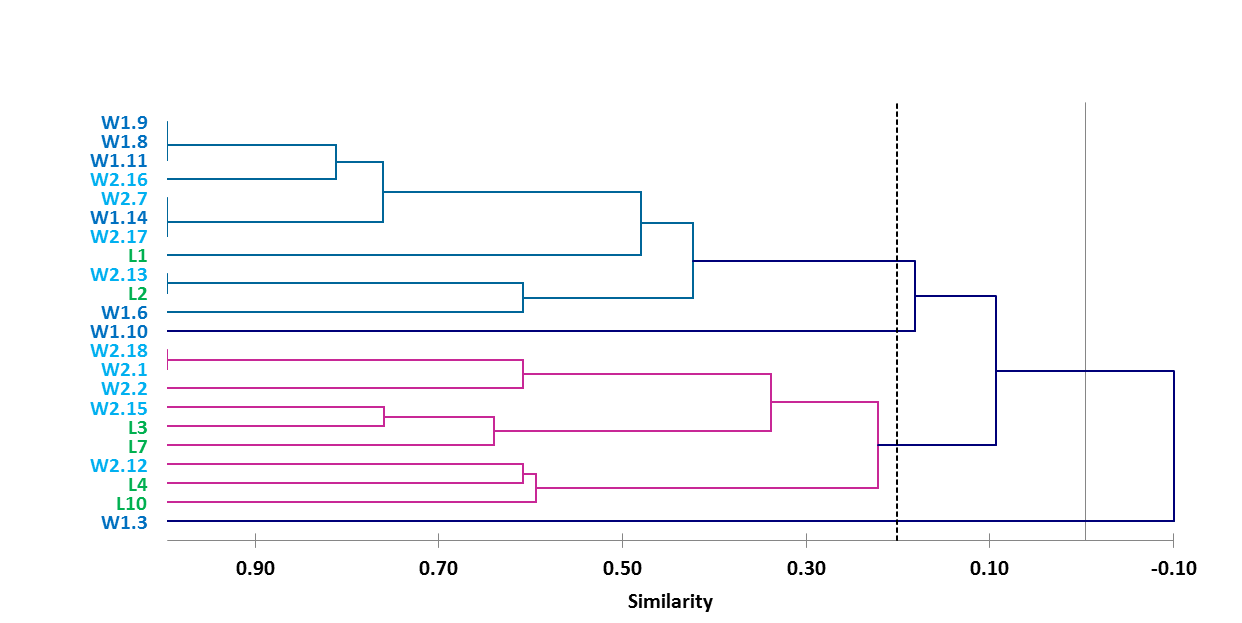


**W2- Lettuce irrigation water; W1- Second water source; L- Lettuce**

**Supplementary Figure 1.**

Supplement: Supplementary file 1 [file mbt20008-0462-sd1.doc]
